# North‐facing slopes and elevation shape asymmetric genetic structure in the range‐restricted salamander *Plethodon shenandoah*

**DOI:** 10.1002/ece3.5064

**Published:** 2019-04-16

**Authors:** Kevin P. Mulder, Nandadevi Cortes‐Rodriguez, Evan H. Campbell Grant, Adrianne Brand, Robert C. Fleischer

**Affiliations:** ^1^ Center for Conservation Genomics, National Zoological Park Smithsonian Conservation Biology Institute Washington District of Columbia; ^2^ Research Center in Biodiversity and Genetic Resources CIBIO/InBIO Vairão Portugal; ^3^ Departamento de Biologia Faculdade de Ciências da Universidade do Porto Porto Portugal; ^4^ Department of Vertebrate Zoology, National Museum of Natural History Smithsonian Institution Washington District of Columbia; ^5^ Department of Biology Ithaca College Ithaca New York; ^6^ United States Geological Survey, Patuxent Wildlife Research Center SO Conte Anadromous Fish Research Lab Turners Falls Massachusetts

**Keywords:** amphibians, gene flow, landscape genetics, mitochondrial genome, Plethodontidae, single nucleotide polymorphisms

## Abstract

Species with narrow environmental tolerances are often distributed within fragmented patches of suitable habitat, and dispersal among these subpopulations can be difficult to directly observe. Genetic data can help quantify gene flow between localities, which is especially important for vulnerable species with a disjunct range. The Shenandoah salamander (*Plethodon shenandoah*) is a federally endangered species known only from three mountaintops in Virginia, USA. To reconstruct the evolutionary history and population connectivity of this species, we generated both mitochondrial and nuclear data using sequence capture from individuals collected across all three mountaintops. Applying population and landscape genetic methods, we found strong population structure that was independent of geographic distance. Both the nuclear markers and mitochondrial genomes indicated a deep split between the most southern population and the genetically similar central and northern populations. Although there was some mitochondrial haplotype‐splitting between the central and northern populations, there was admixture in nuclear markers. This is indicative of either a recent split or current male‐biased dispersal among mountain isolates. Models of landscape resistance found that dispersal across north‐facing slopes at mid‐elevation levels best explain the observed genetic structure among populations. These unexpected results highlight the importance of incorporating landscape features in understanding and predicting the movement and fragmentation of this range‐restricted salamander species across space.

## 1 INTRODUCTION

The theory of island biogeography has been applied to montane‐adapted species when patches of suitable habitat (“sky islands”) are surrounded by areas of unsuitable or impassible lowlands (Brown, 1971; MacArthur & Wilson, 1967). Mountain top species share many of the same characteristics as island species, including relatively small population sizes, fragmented geographic ranges, and specialized ecological adaptations (Brown, 1971; McCormack, Huang, Knowles, Gillespie, & Clague, 2009). Because of their narrow physiological tolerances and limited dispersal ability (Velo‐Antõn, Parra, Parra‐Olea, & Zamudio, 2013), montane endemics are also predicted to be at increased risk of climate‐induced local extirpation, leading to extinction (Bernardo & Spotila, 2006; Walls, 2009). Unlike most island model systems, however, montane ecotones are typically less abrupt, with populations separated by gradual changes in habitat suitability (McCormack et al., 2009). These ecosystems and species can thus be useful systems in which to explore the climatic and landscape features that may influence dispersal, migration, and evolutionary history for species with low vagility, such as salamanders.

**Figure 1:**
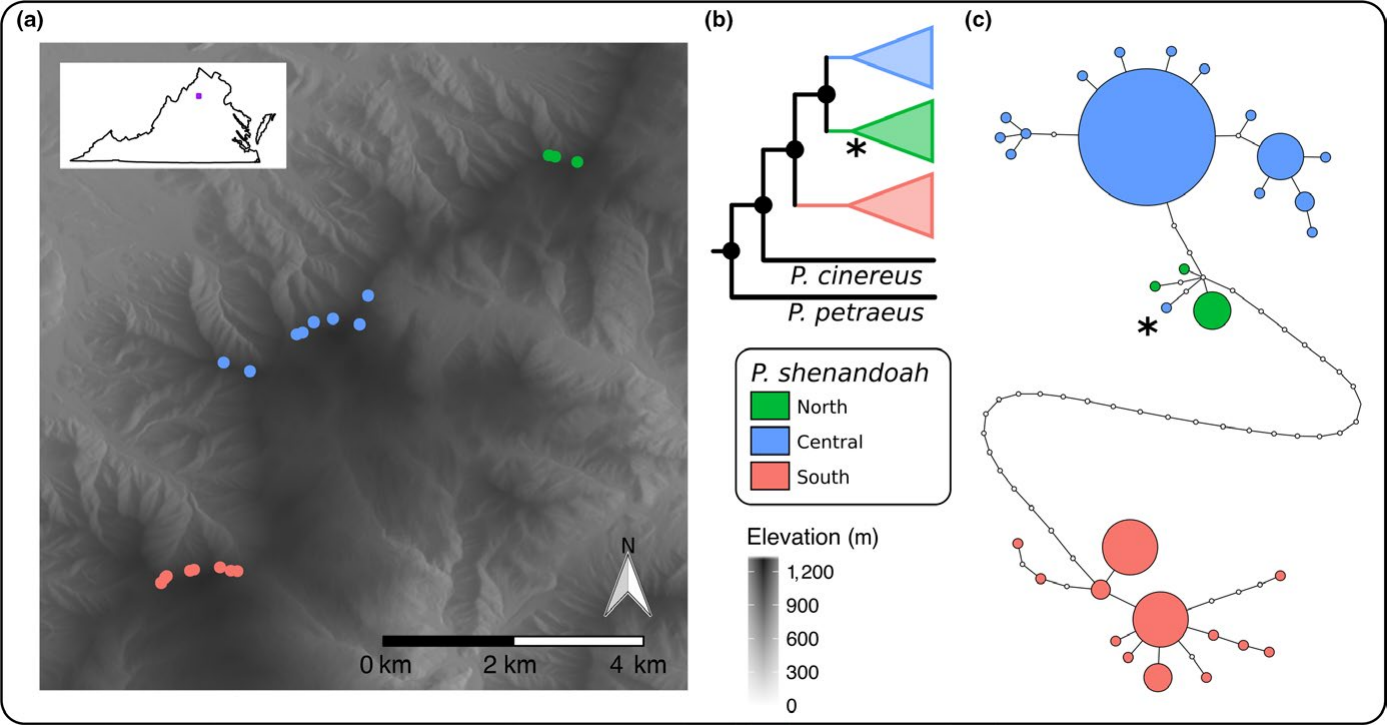
(a) Shaded relief map of our study area in Shenandoah National Park. Genetic sampling localities are indicated by dots and colored by mountain and cover the complete known distribution of *P. shenandoah*. Inset shows the location of the study area in the state of Virginia, USA. (b) Phylogenetic reconstruction based on 78 *P. shenandoah* samples and two outgroup species. Nodes with a posterior probability above 0.99 indicated by black dots and all shallow nodes were collapsed for viewing. (c) Haplotype network of 67 *P. shenandoah* samples that had over 90% of the target mitochondrial genome covered. Asterisk denotes a single individual from the central population that grouped with the northern samples in both the haplotype network and the tree

The Appalachian Mountains are a biodiversity hotspot for lungless salamanders (Plethodontidae; Petranka, 2010; Rissler & Smith, 2010) and biodiversity peaks at mid‐elevations (~900 m; Kozak & Wiens, 2010). Terrestrial *Plethodon* salamanders are associated with cool, moist microhabitats in mature, upland forests where temperature and moisture conditions allow for cutaneous respiration and successful foraging of invertebrates. Because *Plethodon* salamanders are lungless and rely on cutaneous respiration, individuals typically demonstrate avoidance of warmer temperatures and drier substrates (Peterman & Semlitsch, 2014; Riddell & Sears, 2015). Widespread *Plethodon* such as the red‐backed salamander (*Plethodon cinereus)* have small home ranges (males ~ 50–75 m^2^, females ~ 5–10 m^2^; Muñoz, Miller, Sutherland, & Grant, 2016), low fecundity, and limited, male‐biased dispersal (Liebgold, Brodie, & Cabe, 2011). A sister species to the red‐backed salamander (Highton, 1995), the Shenandoah salamander (*Plethodon shenandoah*) is a montane‐adapted species that is federally endangered in the US and is considered Vulnerable by the International Union for Conservation of Nature (Hammerson & Mitchell, 2018). Populations of *P. shenandoah* are restricted to three mountaintops in Shenandoah National Park (Figure 1a). In order to safeguard the exact locations of the species, we refer to the studied localities as North (N), Central (C) and South (S) (see Figure 1a). The species primarily occurs on north‐facing slopes above 850 m, although its distribution may have been more widespread during the cooler periods of the Pleistocene (Jaeger, 1970). The fragmented range is hypothesized to be a result of the narrow suitability of current climatic and microhabitat conditions, and potential competition with *P. cinereus*(Dallalio, Brand, & Grant, 2017; Highton & Worthington, 1967). In order to manage and conserve *P. shenandoah*, the National Park Service needs to better understand the evolutionary history of the species, define current population boundaries, and identify the potential landscape features that mediate among‐population dispersal (USFWS, 1994). This will also contribute to our understanding of the factors that impact the movement of salamanders, the effects of fragmented habitats on genetic variation, and help managers consider the influence of landscape features in the management of montane ecosystems in the central Appalachian Mountains.

For many species with low vagility and cryptic behaviors, directly observing dispersal and population connectivity is difficult; therefore, we rely on genetic data to quantify gene flow and identify barriers to migration. Previous genetic work across the range of *P. shenandoah*found only three haplotypes within 404 basepairs (bp) of the mitochondrial Cytochrome‐*b* gene, and failed to find population structure between mountains, indicating either contemporary gene flow or recent population fragmentation (Carpenter, Jung, & Sites, 2001). A single mitochondrial fragment may, however, lack the resolution of the full mitochondrial genome (Yu, Li, Ryder, & Zhang, 2007). In addition, mitochondrial markers only reflect maternal gene flow and cannot detect male‐biased dispersal (Liebgold et al., 2011). For this study, we greatly expanded on the genetic sampling by using an in‐solution sequence capture approach to enrich both nuclear markers and near‐complete mitochondrial genomes. This enabled us to accurately re‐construct the evolutionary history of the species, identify genetic structure across the three mountaintop populations of its range, and apply landscape genetic methods to identify potentially important barriers to gene flow.

## 2 MATERIALS AND METHODS

### 2.1 Study area and sampling

In 2012–2013, we collected tail tips of *P. shenandoah* across all three mountains where the species is known to occur. Areas between known localities were also searched for potential dispersers or unknown populations, but no Shenandoah salamanders were detected outside of these three mountaintops and we believe we have captured the entirety of the species' range in our sampling (Figure 1a). Salamanders were found by turning natural cover objects within 32 × 2 m transects and sampled by taking ~0.5 cm of tail tissue using sterile forceps to induce tail autotomy. Tail tips were stored in 96% ethanol and all individuals were photographed, measured, and subsequently released at their capture location.

### 2.2 Sequence array design and laboratory methods

To sequence the same subset of loci across all our samples we performed sequence capture using a synthetic probe array from MYcroarray (Ann Harbor, Michigan, USA) to enrich our genomic libraries for a predetermined set of loci. As no genomic or transcriptomic data were available for *Plethodon*salamanders at the time, we generated genomic data for probe design by sequencing five samples of *P. shenandoah* and five of its sister species *P. cinereus* by means of RADtag sequencing on two MiSeq lanes. Mitochondrial genomes were reconstructed from bycatch sequences and putative nuclear single nucleotide polymorphisms (SNPs) were identified at both the species and population levels. We focused on developing a probe set appropriate for both species to facilitate future work. We targeted 875 loci based on the RADtag sequences, added 334 partial UCE's that had previously worked on *Psuedotriton ruber* (personal communication Brant Faircloth) and 43 conserved loci that were found in publicly available transcriptome assemblies of *Ambystoma mexicanum* and *Notophthalmus viridescens* (Abdullayev, Kirkham, Björklund, Simon, & Sandberg, 2013; Wu, Tsai, Ho, Chen, & Lee, 2013). Our final probe set targeted a total of 1,253 nuclear loci as well as a ~15 kb region of the mitochondrial genome (see Supporting Information for further details on probe design).

Genomic DNA was extracted from 85 *P. shenandoah* samples using the Qiagen DNeasy blood and tissue kit (Valencia, CA, USA) following manufacturer's guidelines. Up to 2,000 ng of gDNA was sheared to an average size of ~400 bp using the Q800R sonicator (QSonica, Newton, Connecticut, USA) and subsequently prepared for Illumina sequencing using Nextera‐style adapters with dual‐indexed 8 bp barcodes. Libraries were quantified by Qubit 2.0 and samples of similar concentrations were pooled in groups of 6–10 samples for a total of either 500 ng or 1,000 ng in 3.4 μl of volume. Hybridization followed manufacturer's guidelines for MYcroarray's stringent protocol except for the increased hybridization time of 48 hr as well as an increased concentration of Cot‐1. Following sequence capture, the samples were re‐amplified for 16 cycles using Nextera primers, quantified using qPCR and sequenced with paired‐end reads of 178 bp and 151 bp on two lanes of HiSeq 2500.

### 2.3 Bioinformatic processing

We demultiplexed sequences, removed adapters with Cutadapt 1.9 (Martin, 2011), and mapped reads against the partial mitochondrial genome with bowtie 2.3 (Langmead & Salzberg, 2012), using strict end‐to‐end mapping. Only reads where both pairs mapped concordantly were kept, and PCR duplicates were removed with Picard 2.5. Files were imported into Geneious 10.2.2 (Kearse et al., 2012), manually checked for inconsistencies and consensus sequences were called requiring a minimum depth of five unique reads, and trimmed to the length of the original reference sequence. Any sample that showed signal for multiple haplotypes or had less than 10% of the mitochondrial genome covered was removed at this stage due to potential contamination or lack of signal, and not included for further analyses or nuclear SNP calling.

Due to the small size of the targeted loci (100 bp) and the large size of the salamander genome, using only the baits as a reference sequence may result in paralogs being combined as one locus, resulting in spurious SNPs. To create a reference of longer contigs that could distinguish between similar loci, we assembled all the reads that did not map to the mitogenome for three individuals of each species (*P. shenandoah* and *P. cinereus*) together with Trinity v2.4 using default settings (Haas et al., 2013). Reads for all individuals were mapped against the reference sequence using bowtie 2.3, not allowing for insertions and deletions and with strict mapping to reduce the chance of paralogs mapping to the same location (‐‐mp 48). Reads that did not map concordantly were removed with samtools 1.3.1 and duplicates were removed with Picardtools. SNPs were called using GATK v3.7 using the HaplotypeCaller pipeline (McKenna et al., 2010). We generated a dataset of bi‐allelic SNPs with low quality SNPs removed using vcftools 0.1.15 (‐‐minQ 30 ‐‐minDP 3 ‐‐mac 2 ‐‐non‐ref‐ac 6).

**Figure 2:**
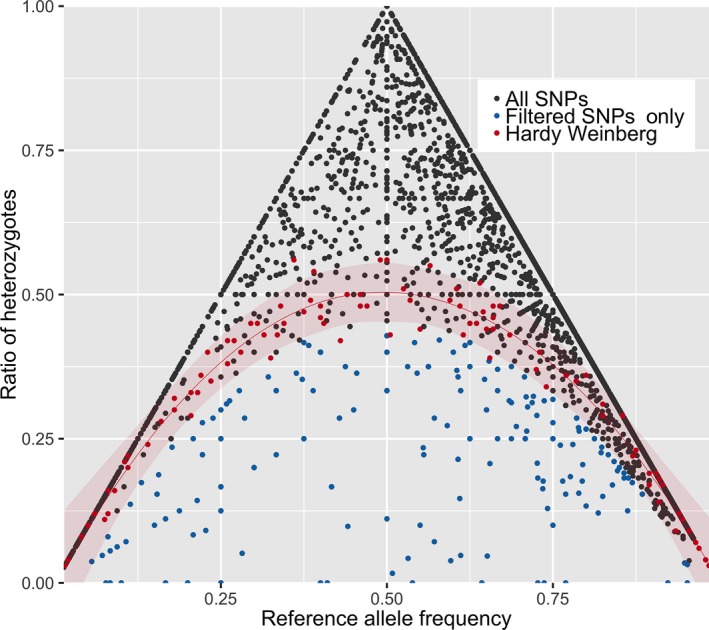
A graphical representation of the interaction between allele frequency of *P. shenandoah* samples and the ratio of heterozygotes for all SNPs. The *x*‐axis is the allele frequency of the reference allele and the y‐axis is the ratio of heterozygotes for that locus. We also added a simulated population in Hardy Weinberg equilibrium (HWE) in red for comparative purposes. All SNPs are plotted for both the initial dataset of all SNPs and the final heterozygosity filtered dataset. The triangle shape is an inert aspect of the fact that the maximum ratio of heterozygotes is dependent on the allele frequency of that locus

In order to further filter our SNPs for potential paralogs, we calculated observed (Ho) and expected heterozygosity (He) for every SNP with plink v1.05 (Danecek et al., 2011). To visualize and compare the SNPs we also simulated a population of 100 SNPs in Hardy Weinberg Equilibrium (HWE) using the HardyWeinberg 1.5.8 R package. All SNPs were plotted in a scatterplot using ggplot 2.2.2.1 with the reference allele on the *x*‐axis and observed heterozygosity on the *y*‐axis (Figure 2; custom Rscripts available on request from KPM). Our final nuclear SNP dataset consisted of the subset of SNPs for which the observed heterozygosity was lower than the expected heterozygosity in order to remove SNPs that are more likely to be paralogs. Samples that had less than 15% of the nuclear SNPs genotyped were removed from both nuclear and mitochondrial datasets. All subsequent nuclear analyses were performed on both the initial quality filtered SNP dataset as well as the final paralog filtered dataset but we only report the final dataset here.

### 2.4 Phylogenetic reconstruction

Mitochondrial consensus sequences passing all quality controls were aligned using MUSCLE v3.8.1551 (Edgar, 2004) using a maximum of 16 iterations and including two outgroups from GenBank; the sister species *P. cinereus* (AY728232) and *P. petraeus* (AY728222). For phylogenetic reconstruction, we removed all non‐coding sequences and partitioned the data by codon position for a total alignment of 11,310 bp. We estimated the phylogeny and divergence times using Bayesian inference in BEAST 2.5.0 (Bouckaert et al., 2014) applying a constant coalescent population and a strict molecular clock with a normal distribution centered at a divergence rate of 0.02 per Myr (95% HDP = 0.012–0.0298; Crawford, 2003). We set a prior for the MRCA of all samples at 27 Mya based on the age of the eastern plethodontid clade that includes *P. petraeus* (Kozak, Weisrock, & Larson, 2006). Substitution models were allowed to vary by codon position and were estimated during the BEAST run with bModelTest 1.1.0 (Bouckaert & Drummond, 2017). The MCMC was run twice for 10 million generations sampling every 5,000 generations. We discarded 10% as burn‐in and built a maximum clade credibility tree using TreeAnnotator. The tree was edited for publication with iTol v3 (Letunic & Bork, 2016) and Inkscape 0.17. A subset of samples that had over 90% of the reference covered (coding and non‐coding) was aligned independently and used to build a haplotype network using TCS 1.21 with gaps represented as a 5th state (Clement, Posada, & Crandall, 2000). Networks were imported into tcsBU to add population labels and create the figures (Múrias Dos Santos, Cabezas, Tavares, Xavier, & Branco, 2015).

### 2.5 Population genetic analyses

We used fastStructure with a simple prior using the wrapper scripts of structure‐threader to explore up to seven different levels of K and identify the value of K that maximized marginal likelihood (Pina‐Martins, Silva, Fino, & Paulo, 2017; Raj, Stephens, & Pritchard, 2014). This *K* value was subsequently run using a logistic prior and 500 cross‐validation tests for increased sensitivity for the final dataset. Plots of the Qmean values were constructed using R package strataG 2.0.2 (Archer, Adams, & Schneiders, 2017). Genetic population differentiation between mountains was calculated using the R package diveRsity 1.9.90 (Keenan, Mcginnity, Cross, Crozier, & Prodöhl, 2013). Pairwise *F*_ST_ values between populations are reported in the results as this differentiation measure is widely used and is considered an appropriate measurement for bi‐allelic SNP markers (Meirmans & Hedrick, 2011).

### 2.6 Landscape genetic analyses

To explore possible landscape variables that contribute to genetic structure, we created eight resistance surfaces (Supporting Information Figure S2) that we hypothesized could impact movement of salamanders on the landscape. Slope, Topographic Position Index (TPI, using eight neighbors), roughness, northness, westness, and Topographic Wetness Index (TWI) were all derived from a 15 m DEM using the R packages elevatr 0.1.4, raster 2.6.7, spatialEco1.1.0 and dynatopmodel 1.2.1 (Evans, 2017; Hijmans, 2017; Hollister & Shah, 2017; Metcalfe, Beven, & Freer, 2018). Solar incidence was derived from the same 15 m DEM using ArcGIS 10.3 (ESRI, Redlands, CA, USA) and the Normalized Difference Vegetation Index (NDVI) was calculated using bands 4 and 5 from cloudless Landsat8 Images taken in May 2015. All surfaces were rescaled to 0–100 and inverted if necessary to have higher values represent higher expected landscape resistance (see Supporting Information Table S1 for a detailed description). We also generated a separate set of surfaces based on the same landscape variables but restricted to elevations above 500 m to generate more biologically relevant dispersal paths for *P. shenandoah* (Supporting Information Figure S3). This elevational cut‐off was chosen as it allows potential dispersal pathways to pass through lower elevations outside the current elevational limits of ~850 m; this cut‐off value also corresponds to an elevation at which the base resistance (all resistance values = 1) as calculated by CIRCUITSCAPE v 4.0.5 (McRae & Shah, 2009), is close to equal between the transition from Mountain N to C and from Mountain C to S. This base resistance can differ for non‐uniform surfaces when parts of the raster surface are removed (see Figure 4) and by ensuring that base‐resistances are equal between both transitions, we do not have to correct for base resistance in all other resistance surfaces.

The Shenandoah salamander primarily occurs between 850–1,050 m, but can also be found infrequently up to the highest elevation at the peaks (1,230 m), thus we did not expect that a simple linear response would adequately represent dispersal resistance across elevations. We, therefore, created multiple surfaces along nine different elevations from 800 to 1,200 m in 50 m intervals using a Gaussian transformation of the 15 m DEM using ArcGIS (Supporting Information Figure S4). These represent resistance surfaces that would capture optimal climatic or topographic factors that facilitate dispersal along intermediate elevations.

We used CIRCUITSCAPE (McRae & Shah, 2009) to evaluate each resistance surface and calculate the mean resistance between centroids of all genetic sample locations for each mountain. The Euclidian distance among centroids of *P. shenandoah* populations is roughly the same, as Mountains N and S occur ~5 km to the northeast and southwest, respectively, of Mountain C. Distance is also incorporated into the resistance measure calculated by CIRCUITSCAPE, so we did not include topographic distance separately as a factor influencing genetic structure. Landscape genetic theory has recently developed several methods to compare genetic distances between populations with resistance values to statistically identify significant landscape features that are important in explaining the genetic structure of a study system. Multiple matrix regression with randomization (MMRR) can be used to rank the importance of resistance surfaces (Wang, 2013), but requires large sample sizes when evaluating multiple landscape features. Resistance GA optimizes resistance surfaces using genetic distances but is also reliant on multiple comparisons in order to reduce type I error (Peterman, 2018). Neither of these methods is applicable to our study system as *P. shenandoah* is restricted to three populations and thus three comparisons; therefore, we limit our analyses to a descriptive comparison and discussion of different resistance surfaces relevant to the habitat and biology of Shenandoah salamanders.

## 3 RESULTS

### 3.1 Phylogenetic reconstruction

Seventy‐eight *P. shenandoah* samples had sufficient coverage in both mitochondrial and nuclear markers to be included for further analyses (32 for the southern mountain, 40 for the central and 6 for northern). The mitochondrial phylogenetic tree (Figure 1b) supports a sister relationship between *P. shenandoah* and *P. cinereus* with the divergence between the species estimated at ~8.5 mya (HPD: 7.5–9.5). Within *P. shenandoah* we recovered three lineages corresponding to the three mountains with an earlier divergence between the southern population and the other two (220 kya, HPD: 149–290) and more recent divergence between the northern and central populations (74 kya, HPD: 44–105). A total of 67 Shenandoah samples had over 90% of the selected mitochondrial genome covered and were included in the haplotype network (GenBank MK493161–MK493192). The haplotype network (Figure 1c) recovers fewer steps (substitutions) linking the northern and central haplotype groups than in comparison to the southern group. The single central population individual that grouped with the northern haplotypes (asterisk in Figure 1c) was also part of the northern clade in the phylogenetic tree (asterisk in Figure 1b). To confirm these results, we re‐extracted and re‐sequenced this individual using Sanger sequencing for a 358 bp segment of the ATP6 gene with known differences between haplotype groups (see Supporting Information for details). This analysis supported the placement of this central population individual with the northern haplotypes.

### 3.2 Population genetics

Following mapping of reads to the Trinity reference sequence and stringent SNP filtering, we obtained 4,640 SNPs. When plotting minor allele frequency versus the level of heterozygosity (Figure 2), many of these loci had strong support as a SNP but a large number were possible paralogs. Following very strict filtering of all SNPs where observed heterozygosity was higher than expected heterozygosity (Ho > He) resulted in a final dataset of 222 SNPs (blue dots in Figure 2).

**Figure 3:**
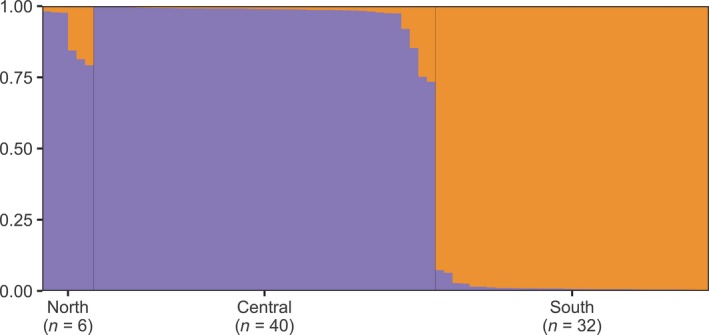
FastStructure plot of 222 nuclear SNPs from 78 *P. shenandoah* samples across three populations run with a logistic prior and 500 repetitions at *K* = 2. Every vertical bar represents an individual and the colors indicate the proportion assigned to either of the two groups identified by FastStructure. Black lines separate the geographic localities. There is a clear split between the southern population and the central and northern populations

FastStructure identified *K* = 2 as the model that maximizes the marginal likelihood. The two clusters correspond to the northern and central populations forming one group, and the remaining samples belonging to the southern cluster. These results held whether we used the final small dataset (Figure 3) or initial large dataset (that may have contained paralogous loci, see Supporting Information Figure S1). We recovered the same pattern of divergence in the *F*_ST_ calculations with higher values for the split between the southern population and the two others (Table 1). The overall high *F*_ST_ values for the final filtered dataset compared to the initial dataset are likely due to our low heterozygosity filtering.

**Table 1:** *F*_ST_ genetic differentiation values between all mountains split by both the initial larger dataset of 4,640 SNPs and the final dataset which was filtered for heterozygosity with 222 SNPs

	CN	CS	NS
Initial dataset	0.0103	0.0466	0.0408
Final dataset	0.0729	0.2585	0.2369

### 3.3 Landscape genetics

**Figure 4:**
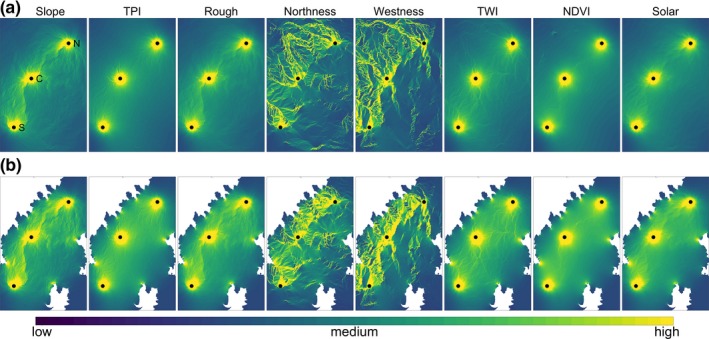
CIRCUITSCAPE results for eight landscape surfaces that we investigated for correlation with observed genetic distance among populations of *P. shenandoah*; Slope, Topographic Position Index (TPI), Roughness, Northness, Westness, Topographic Wetness Index (TWI), Normalized Difference Vegetation Index (NDVI), and Solar Incidence (Solar). (a) The current maps when using the full extent and (b) shows the resistance values when restricting CIRCUITSCAPE to only run at elevations above 500 m to avoid creation of corridors outside the known elevation limits of *P. shenandoah*

Although the geographic distances between the Central mountain and the Southern mountain (henceforth called the southern corridor) and the Central mountain and the Northern mountain (the northern corridor) were almost equal, the mean resistance between populations differed for most of the landscape resistance surfaces we examined. In all cases, resistance was substantially higher when calculated directly between the Southern and Northern mountain (the complete corridor), making a stepwise model of dispersal more likely (see Supporting Information Table S2). This was often also apparent from the modeled currents, which mostly travelled over or near to the Central mountain (Figure 4). The least resistance currents for some of the landscape surfaces crossed substantially into the low elevation areas of the raster when allowing CIRCUITSCAPE to travel the full extent of the map, which we considered to be ecologically unlikely. When restricting the current to elevations above 500 m, the main current paths more closely followed the ecologically relevant paths along the mountain chain (e.g., see Figure 4b, northness and westness).

**Figure 5:**
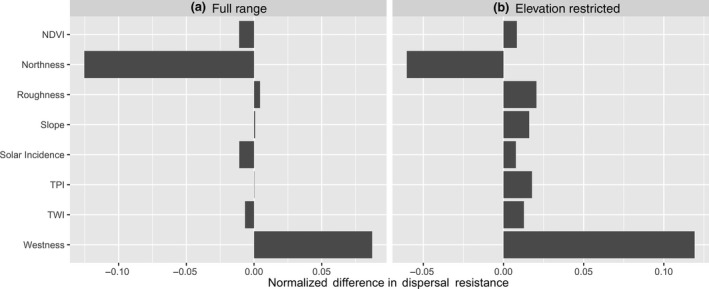
Boxplot of the normalized difference in mean resistance values between the southern and northern corridor between *P. shenandoah* populations as calculated by CIRCUITSCAPE. Values below zero indicate lower relative resistance values for the northern corridor. (a) The values when applying the resistance model to the full extent of the range (see also Figure 4a), (b) When restricting it to only elevations above 500 m. The northern corridor shows lower resistance for NDVI, northness, Solar Incidence and TWI when using the full map and only shows lower resistance for northness when restricted to paths above 500 m

Across all landscape surfaces, resistance levels were mostly higher for the northern corridor than the southern corridor, especially for the elevation‐restricted rasters. If these landscape features are a good reflection of realized dispersal, then the direction and magnitude of resistance should correlate with the genetic structure of genetic divergence (Table 1). Four resistance surfaces (NDVI, northness, solar incidence, and TWI) showed a pattern in the same direction with lower values for the northern corridor compared to the southern corridor, but only northness showed a considerable difference between the two (Figure 5a). In the elevation‐restricted dataset, only northness correlated with the genetic results (Figure 5b).

**Figure 6:**
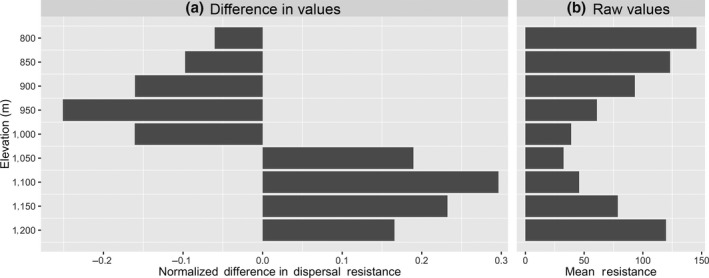
(a) Boxplot of the normalized difference in mean resistance among *P. shenandoah* populations for nine different elevational surfaces as calculated by CIRCUITSCAPE. Resistance for the northern corridor is much lower along the lower elevations (800–1000m) than at higher elevations. (b) Mean combined resistance values for both corridors, indicating that general resistance is lower along mid‐elevations regardless of the corridor

The maximum elevation on Mountain N is approximately 1,100 m whereas the highest points on Mountains C and S are greater than 1,200 m. For this reason, the relative landscape resistance for the northern corridor was lower from 800–1,000 m and much higher between 1,050–1,200 m (Figure 6). The lower elevation levels are thus consistent with the genetic structure results that combine Mountain N and C in one genetic deme, and also consistent with the elevations at which *P. shenandoah* is found most frequently (800–1,050 m).

## 4 DISCUSSION

Despite the small geographic distances among mountains, we found a strong and consistent signal of genetic structure in both the nuclear and mitochondrial datasets for *P. shenandoah*. Given similarity in geographic distances among the populations, we hypothesized that *P. shenandoah* populations would have a similar genetic distance among the three mountains. However, from both the mitochondrial and nuclear DNA results, we identified a strong split between the more similar northern and central populations and the distinct southern population.

Our results contrast markedly with the only other population genetics study on the Shenandoah salamander, which found no mitochondrial genetic structure across the three mountains (Carpenter et al., 2001). The Carpenter et al. (2001) study was based on 28 individuals sequenced for 404 bp of the mitochondrial Cytochrome‐b marker that resulted in three different haplotypes; AF302894 specific to Mountain C, AF302877 specific to Mountain S and AF302875 found on all three mountains (haplotype names correspond to GenBank accession numbers). The comparison to our larger dataset of 32 unique haplotypes across 78 individuals shows that the targeted 404 bp cannot distinguish between the majority of haplotypes, and therefore, fails to reveal the genetic structuring we find using a greater proportion of the mitochondrial genome. The addition of genome‐wide markers also showed the corresponding signal of the nuclear genome which was not clear from the original allozyme data in Carpenter et al. (2001). This disparity shows the importance of sequencing nuclear markers in addition to considering a larger part of the mitogenome for species with small isolated populations that may not have much genetic variation.

The estimated divergence date between *P. cinereus* and *P. shenandoah* based on the mitochondrial genome was ~8.5 mya which is marginally earlier than previous studies estimating this split at ~7.5 mya (Wiens, Engstrom, & Chippindale, 2006) and ~6 mya (Kozak et al., 2006). Although the confidence intervals are large and phylogeographic datasets can overestimate node age (Ho, Phillips, Cooper, & Drummond, 2005), we estimate that the southern population split from the other two around 220 kya which would roughly correspond to Highton's hypothesis that the species was once more widespread and that the populations were connected during the Pleistocene (Highton & Worthington, 1967). There was some structuring of mitochondrial haplotypes between the northern and central population (see Figure 1c), but we detected no significant signal of genetic structure in the nuclear genome. We also found one mitochondrial haplotype that grouped with the northern samples but that individual was found on the northern edge of the central range. Taken together, these results suggest these populations either split recently or that some gene flow and dispersal, though rarely detected, is ongoing. *Plethodon cinereus* readily disperses across short distances of up to 90 m when displaced or when new habitat is available (Kleeberger & Werner, 1982; Marsh, Thakur, Bulka, & Clarke, 2004) and there are incidental reports such as a single female moving >140m (Sterrett, Brand, Fields, Katz, & Grant, 2015). Rare long‐distance events are difficult to document and largely unknown for plethodontids, but have been shown to influence population processes in other amphibians (Smith & Green, 2006), especially if the species is not dependent on discrete waterbodies for reproduction (Measey, Galbusera, Breyne, & Matthysen, 2007).

The discordance we detected between the nuclear and mitochondrial markers could be the result of male‐biased dispersal, which would remove genetic structure in the nuclear genome while maintaining mitochondrial structure (Toews & Brelsford, 2012). Sex‐biased dispersal is documented in red‐backed salamanders, which have greater philopatry in females and higher male‐biased natal dispersal, based on both genetic and mark‐recapture data (Liebgold et al., 2011); indeed the average home range of males is larger than females (Muñoz et al., 2016). These unexpected genetic results highlight that species with cryptic behavior, low vagility, strong physiologic constraints, and high site fidelity, such as the *Plethodon* salamanders studied here, can still exhibit gene flow even between populations that appear fragmented. The large difference between connectivity from the central locality to both neighboring populations show that successful dispersal is not solely governed by geographic distance, but that favorable landscape features play an important role in facilitating long‐distance dispersal.

The only landscape features which were strongly correlated with the genetic distances were elevation and northness, which correspond with the ecological expectation that *P. shenandoah* is currently known to occur primarily on the north‐facing slopes within a narrow elevational range. Differences in slope and aspect are known to influence a wide variety of habitat features such as solar radiation (Kumar, Skidmore, & Knowles, 1997), moisture content (Hanna, Harlan, & Lewis, 1982), temperature (Suggitt et al., 2011), and soil composition (Kutiel, 1992), all which may underlie species community structure (Sternberg & Shoshany, 2001). For the Appalachian Mountains, these differences generally mean a more xeric landscape for south‐facing slopes and more mesic conditions on northerly slopes (Desta, Colbert, Rentch, & Gottschalk, 2004). Lungless salamanders are dependent on moist microhabitats (Peterman & Semlitsch, 2013), and thus northern slopes are generally more favorable and this has been shown to be important in describing salamander abundance in other species such as *Plethodon stormi* (Suzuki, Olson, & Reilly, 2008) and in *Salamandrina*(Romano et al., 2017). Although species abundance and gene flow are not directly related, it is not unreasonable to expect areas with lower abundance to correlate with increased dispersal resistance. Unexpectedly, other landscape features we used to describe hypothesized physiological constraints to movement (rough terrain, steep slopes, solar exposure, low soil moisture) did not correlate with the genetic results, suggesting that these factors may not be limiting.

We also found that resistance across a given elevation was consistently lower between Mountains N and C for the elevations where *P. shenandoah* occurs more frequently (800–1,050 m, Figure 6), likely because constant elevations between localities can facilitate dispersal in montane species (Giordano, Ridenhour, & Storfer, 2007). The difference was mostly because the direct paths for the northern corridor are at intermediate elevation paths, whereas the shortest route for the southern corridor goes over the higher ridge between Mountain C and S (Supporting Information Figure S4), which may serve as a stronger dispersal barrier.

There are several other potential mechanisms that could have produced the observed genetic structure results that are independent of landscape characteristics or gradients. Competition with *P. cinereus* has been thought to be a factor that restricts the extent of the *P. shenandoah* range (Griffis & Jaeger, 1998; Jaeger, 1970). However, the outcome of these interactions may be temperature‐ and moisture‐dependent (Dallalio et al., 2017). If competition is density‐dependent, a difference in competitive pressure from red‐backed salamanders could also have resulted in the observed genetic pattern. This would result in contrasting patterns of genetic variation between both species. Preliminary genetic data of *P. cinereus* in the area also finds a similar pattern of genetic structure between these three mountains (unpublished data) indicating that the structural cause (e.g., landscape) is likely a common denominator. Expanses of south‐facing slopes along the southern corridor would also seem to favor dispersal of *P. shenandoah* if it is indeed more drought tolerant than *P. cinereus* (Jaeger, 1971).

In addition, there are potential factors that we cannot currently test for that could be influencing genetic structure. Recent research finds that the cloud base height sets the lower limit of the distribution of *P. shenandoah*(Grant, Brand, De Wekker, Lee, & Wofford, 2018), so it is possible that climatic factors facilitate transient dispersal differently among populations, which are not well‐described by the large‐scale landscape features thought to structure dispersal via physiologic constraints (Kearney & Porter, 2009). Interestingly, predominant precipitation in the Shenandoah mountains comes from the northwest, which may explain our correlation with population structure and northness. Although unlikely, there could also be undetected populations between the three mountains that could be acting as stepping‐stones for migration connecting the northern corridor better than southern corridor. The observed genetic structure in the species can also be a result of vicariance with gene flow or a more recent colonization event. Although the lower haplotype diversity on Mountain N could be due to a colonization event from Mountain C, we believe that it is most likely due to its smaller population and sample size.

## 5 CONCLUSION

Genetic data collected on three populations of *P. shenandoah* show that northern and central populations group together genetically whereas the southern population is distinct with limited to no gene flow with the other two populations. Phylogenetic reconstruction shows that the southern population likely has been separated from the other two populations since the late Pleistocene. The stronger signal of contemporary gene flow in the nuclear genome is likely a result of male sex‐biased dispersal that allows some mitochondrial haplotype structure across the landscape. Both elevation and north‐facing slopes are potential features that can generate this genetic structure by allowing for dispersal between the central and northern population. These results help us understand the evolutionary history of *P. shenandoah*, and can help guide potential conservation efforts for this endangered and range‐restricted species. Furthermore, our work identifies several landscape features that are likely to influence dispersal for low vagility species such as salamanders and demonstrates the importance of using both mitochondrial and nuclear markers to make population genetic inferences.

## CONFLICT OF INTERESTS

The authors have no conflicts of interest or competing interests to disclose.

## AUTHOR CONTRIBUTIONS

EHCG, AB and RCF conceived and designed the study. AB and EHCG collected the samples and NCR and KPM conducted the labwork. KPM, AB, RCF and EHCG analyzed the data, and wrote the paper. All authors read and approved the final product.

## Data Availability

DNA sequences: GenBank accessions MK493161–MK493192, NCBI SRA: SAMN10922333–SAMN10922410.
