# Supplementary material for: North‐facing slopes and elevation shape asymmetric genetic structure in the range‐restricted salamander Plethodon shenandoah
Source: Ecol Evol. 2019 Apr 16;9(9):5094–105. doi: 10.1002/ece3.5064 (PMC6509443; doi:10.1002/ece3.5064)
Supplement: Supplementary file 1 [file ECE3-9-5094-s001.pdf]

Supplemental Figures

Figure S1. Faststructure plot of the full dataset of nuclear SNPs, run on 78 P. shenandoah samples with a logistic prior and 500 repetitions at K=2. Every vertical bar represents an individual and the colors indicate the proportion assigned to either of the two groups identified by Faststructure. Black lines separate the geographic localities. There is a clear split between the southern population and the central and northern populations.

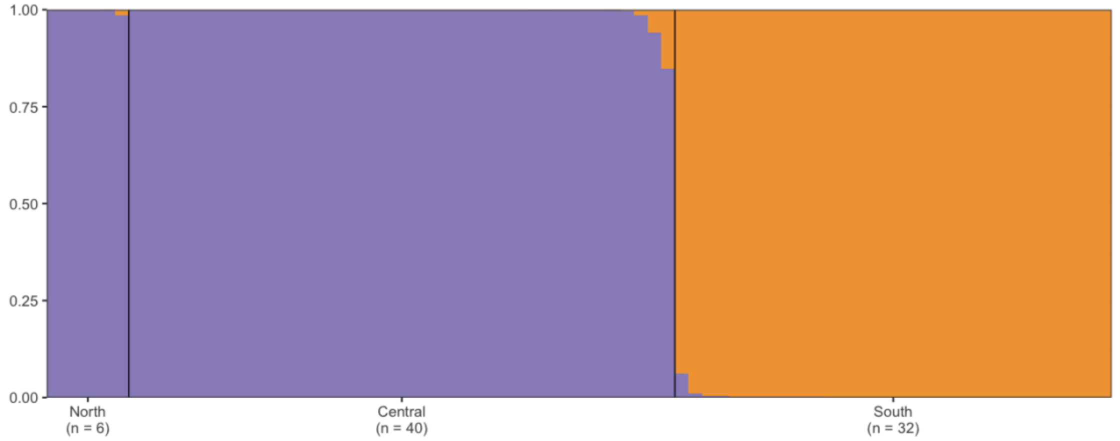

Figure S2. Landscape resistance surfaces for all eight variables at full extent. Lower numbers equal lower resistance and all resistance values are normalized from 0-100. The surfaces include; Slope, Topographic Position Index (TPI), Roughness, Northness, Westness, Topographic Wetness Index (TWI), Normalized Difference Vegetation Index (NDVI), and Solar Incidence (Solar). Locality centers indicated by white dots and named in the first panel.

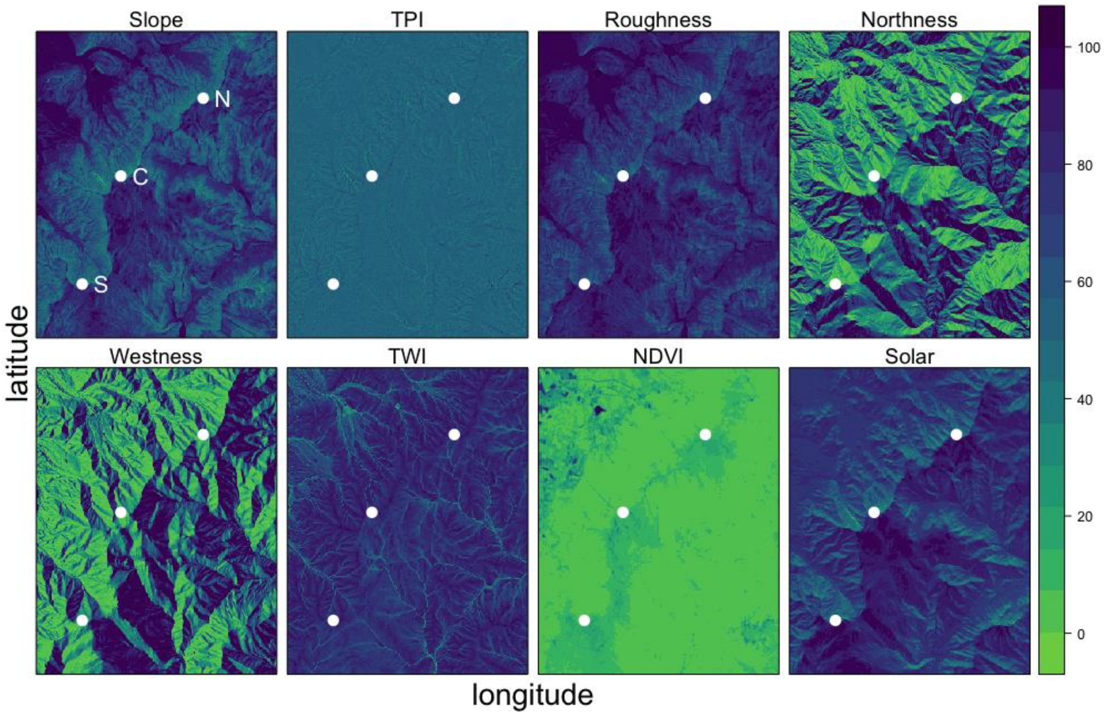

Figure S3: Resistance surfaces for the same landscape variables generated for Figure S2, but restricted for elevation by only allowing cells above 500m. This was done to create more biologically relevant dispersal paths for P. shenandoah. Locality centers indicated by white dots and named in the first panel.

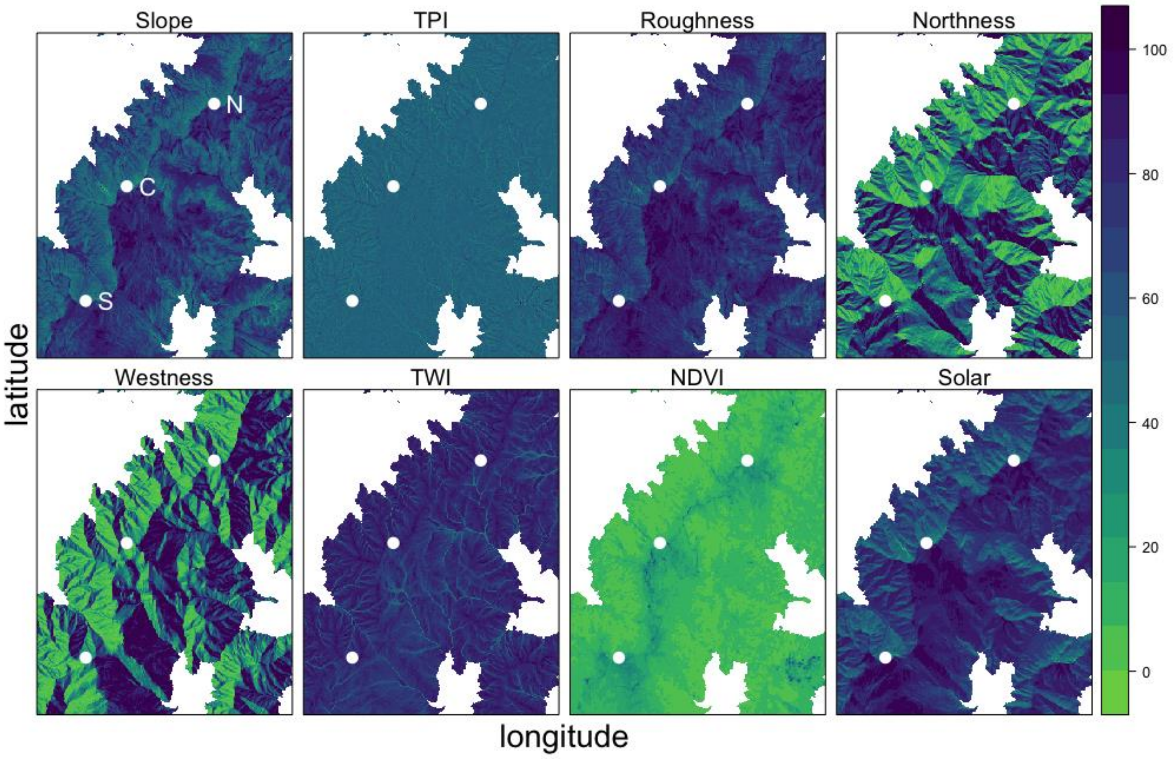

Figure S4: Gaussian transformations of the elevation surface as calculated by ArcGIS 10.3 (ESRI, Redlands, CA, USA) for 9 different mean elevations. Locality centers indicated by white dots and named in the first panel.

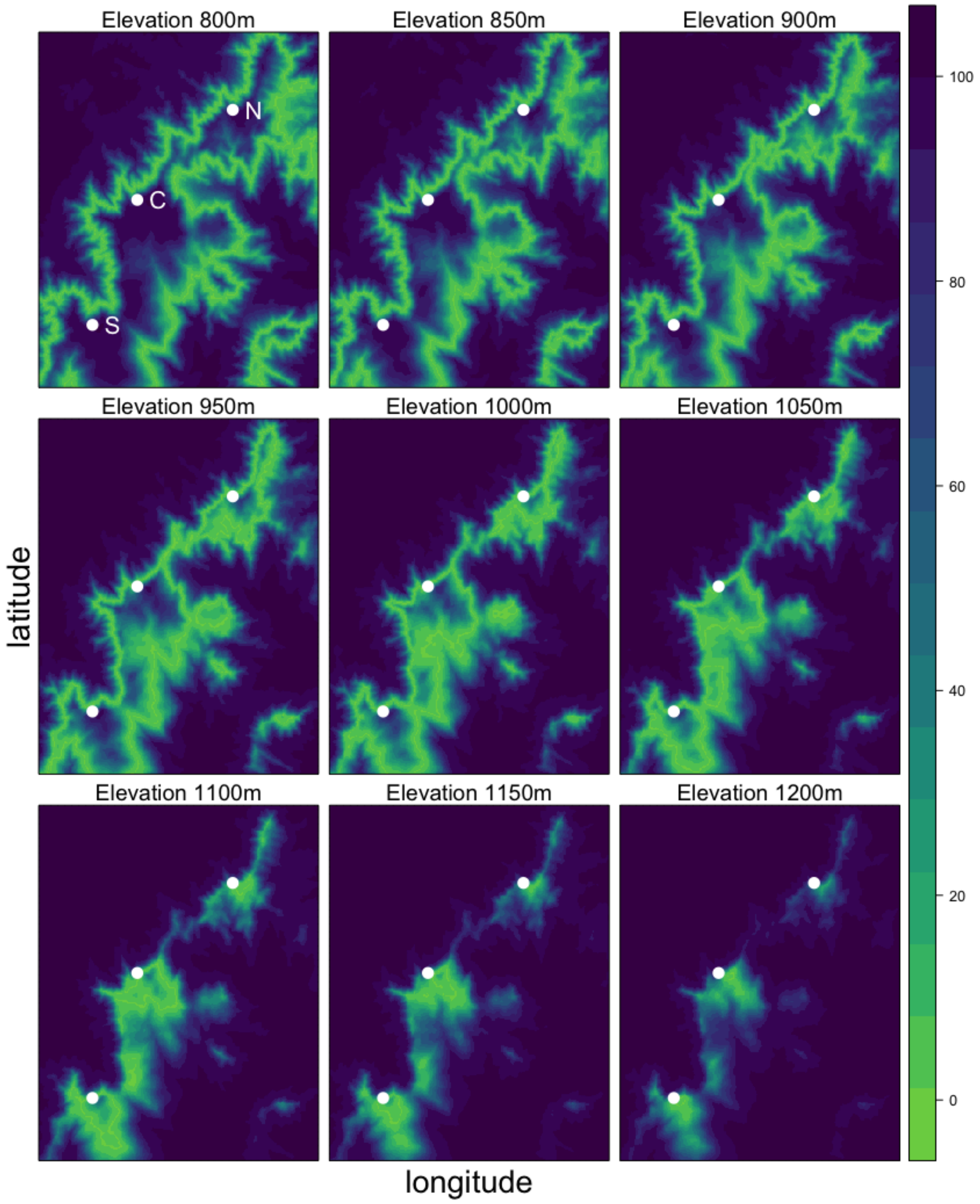

Supplemental Tables

Landscape resistance scores:

All resistance surfaces were scaled from 0-100 and in order to choose the direction of the scale we used a combination of biological knowledge of the species and locality information. We calculated the mean values for all GPS-locations and the 8 surrounding cells and compared that to the mean of all values on the elevation-restricted raster. Unless noted below we chose the direction that would cause the GPS-localities to have a lower resistance value, assuming that individuals would prefer to disperse over similar habitat as they currently inhabit. For some, surfaces values were close and we used biological knowledge of the species to make a final call (see Table S1).

Table S1

**Surface**	**Mean all values**	**Mean GPS localities**	**Invert surface?**	**Reasoning if different**
**Slope**	33.55	48.74	Yes	-
**TPI**	51.19	51.30	No	Difference is very small and according to Peterman and Semlitsch (2013) low TPI scores are ravine like areas and are good for Plethodontid salamanders
**Roughness**	22.73	35.23	Yes	-
**Northness**	49.76	89.34	Yes	-
**Westness**	51.89	36.93	No	-
**TWI**	33.68	29.62	Yes	Plethodontids like wet environments and thus we set lower resistance values for higher wetness scores
**NDVI**	95.49	92.82	Yes	Higher NDVI values relate to greener areas and thus we inversed it
**Solar Incidence**	85.23	72.91	No	-

Table S2. Circuitscape resistance scores for all comparisons, split by full range runs and elevation restricted runs. The difference was calculated by substracting CS from CN and were used to construct Figure 5. CN > Central to North, NS > North to South, CS > Central to South. The following 8 layers were examined: Slope, Topographic Position Index (TPI), Roughness, Northness, Westness, Topographic Wetness Index (TWI), Normalized Difference Vegetation Index (NDVI), and Solar Incidence (Solar).

	**Full Range**				**Elevation restricted to above 500 m**			
**Raster**	**CN**	**NS**	**CS**	**Difference**	**CN**	**NS**	**CS**	**Difference**
Slope	57.63	68.77	57.54	0.001	64.04	81.07	62.02	0.016
TPI	56.24	67.18	56.20	0.000	61.77	77.34	59.62	0.018
Roughness	72.89	87.38	72.24	0.004	80.75	102.23	77.49	0.021
Northness	12.43	19.05	16.00	-0.125	18.88	30.37	21.31	-0.060
Westness	29.14	37.99	24.46	0.087	35.05	48.06	27.58	0.119
TWI	90.76	110.30	91.97	-0.007	99.78	126.55	97.27	0.013
NDVI	100.72	120.97	102.96	-0.011	112.41	141.68	110.56	0.008
Solar	81.14	97.32	82.93	-0.011	90.35	114.53	88.98	0.008

Supplemental Information

Probe development:

We chose five samples each of *Plethodon shenandoah* and *Plethodon cinereus* for RADtag sequencing to develop probes that could both differentiate between species as well as between populations. This included two individuals from Mountain N, and four each from Mountain C and S. Six samples were digested with restriction enzyme *Sbf*I and following the ligation of the first adapter they were sheared to around 300 bp for sequencing. Four samples were digested separately with *Bspq*I and sheared to the same size. Libraries were quantified using qPCR and pooled at equimolar concentrations for sequencing.

Libraries were sequenced on two MiSeq runs at the Dana Farber Cancer institute using paired end 150 bp reads for all 10 samples. We mapped the reads onto two bacterial genomes (*Helicobacter pylori* and *Delftia acidovorans*) to remove potential bacterial contaminant DNA. Resulting sequences were run through stacks 1.05 using default settings (Catchen *et al.*, 2013). As the majority of sequences were not stacking properly we ran assemblies per individual using Geneious 7.0.1 (Kearse *et al.*, 2012). The contigs showed that sequences did align, but not all starting at the same bp as would be expected following a successful restriction enzyme digest. For this reason, we used several different approaches to develop 100 bp capture bait sequences to increase our chances of obtaining valid SNPs.

We developed 12,614 baits using the discoSNP software that finds SNPs during the de Bruijn stage of assembly (Uricaru *et al.*, 2015). A further 1575 baits were developed from the Geneious assemblies by manually finding single SNPs in high quality contigs. All assembled contigs were also blasted to GenBank using Blast+ to identify sequences that had known amphibian annotations and all NON-mitochondrial hits (660 total) were also used. These baits were filtered for overlapping sequences and bad hybridization factors to end up with a total of 10,000 baits that were ordered using the MyBaits set from MycroArray (Ann Harbor, Michigan, USA)

These probes were tested on 63 samples on one HiSeq run to check for enrichment. Following preliminary analyses, we restricted our targets to the 875 loci that showed medium enrichment in order to target only sequences that enriched well but were less likely to be repetitive elements. These 875 loci were supplemented with 334 UCEs, 43 conserved genes and 15kb of the mitochondrial genome (see main text) and were enriched with a new MyBaits probe-set for all samples.

Sanger sequencing:

We developed primers targeting 358 bp of the mitochondrial genome that would be able to differentiate between the three main clades (forward: GGTGTCCTGCYGTTAGGTTTGC, reverse: CATTGACTAGACAGCCGTCC). We amplified representative samples from all three mountains, as well as the individual from Mountain B sample whose mitochondrial haplotype grouped with the mountain C samples. We amplified 2 µl of template DNA using AmpliTaq Gold (Thermo Fisher Scientific, Waltham, Massachusetts) using manufacturers guidelines and 1 µl of each primer diluted to 10nM. We amplified the DNA for 41 cycles, including an initial 6 cycle touchdown from 58C to 52C. All cycles included 30 seconds of 95C, 30 seconds at the annealing temperature and 50 seconds of extension time (72C). The PCR also included an initial denaturation of 5 minutes and a final extension of 10minutes. PCR products were sequenced on an ABI 3130 and analyzed in Geneious 10.2 (Kearse et al., 2012) to determine what clade they belonged to.

Bibliography:

Catchen, J., Hohenlohe, P. A., Bassham, S., Amores, A. and Cresko, W. A. (2013) 'Stacks: An analysis tool set for population genomics', *Molecular Ecology*, 22(11), pp. 3124–3140. doi: 10.1111/mec.12354.

Kearse, M., Moir, R., Wilson, A., Stones-Havas, S., Cheung, M., Sturrock, S., Buxton, S., Cooper, A., Markowitz, S., Duran, C., Thierer, T., Ashton, B., Meintjes, P. and Drummond, A. (2012) 'Geneious Basic: An integrated and extendable desktop software platform for the organization and analysis of sequence data', *Bioinformatics*, 28(12), pp. 1647–1649. doi: 10.1093/bioinformatics/bts199.

Langmead, B. and Salzberg, S. L. (2012) 'Fast gapped-read alignment with Bowtie 2', *Nat Methods*, 9(4), pp. 357–359. doi: 10.1038/nmeth.1923.

Peterman, W. E. and Semlitsch, R. D. (2013) 'Fine-Scale Habitat Associations of a Terrestrial Salamander: The Role of Environmental Gradients and Implications for Population Dynamics', *PLoS ONE*, 8(5). doi: 10.1371/journal.pone.0062184.

Uricaru, R., Rizk, G., Lacroix, V., Quillery, E., Plantard, O., Chikhi, R., Lemaitre, C. and Peterlongo, P. (2015) 'Reference-free detection of isolated SNPs', *Nucleic Acids Research*, 43(2), p. e11. doi: 10.1093/nar/gku1187.
